# Supplementary material for: Limit of detection of Salmonella ser. Enteritidis using culture-based versus culture-independent diagnostic approaches
Source: Microbiol Spectr. 2024 Nov 4;12(12):e01027-24. doi: 10.1128/spectrum.01027-24 (PMC11619426; doi:10.1128/spectrum.01027-24)
Supplement: Supplemental material — Tables S1 to S9; Fig. S1 to S4. [file spectrum.01027-24-s0001.pdf]

AMERICAN SOCIETY FOR MICROBIOLOGY
Journals.ASM.org

# Supplementary Materials

## Supplementary methods

### Access to MFHPB-20 protocol

The MFHPB-20 protocol is available at the following link: https://www.canada.ca/en/health-canada/services/food-nutrition/research-programs-analytical-methods/analytical-methods/compendium-methods/methods-microbiological-analysis-foods-compendium-analytical-methods.html .

To obtain a method/document, select the appropriate link from the Compendium website and an email request will open with the subject line and address already populated. Please do not add or alter any text prior to sending the request. Health Canada's publications office will respond to the request. A PDF format of the document requested will be sent electronically to the originating email address.

### Media recipes

BPW, TBG, RVS, and BGS agar were purchased in powder form and prepared according to manufacturer instructions.

M9 minimal medium (glucose free)

M9 mineral salt solution: 64 g Na2HPO4-7H2O, 15g KH2PO4, 2.5g NaCl, and 5.0g NH4Cl in a total volume of 1000 mL distilled water. Sterilize by autoclaving. At the same time, autoclave bottles of 700 mL and at least 300 mL distilled water. Filter-sterilize

solutions of 1M MgSO4 and 1M CaCl2. M9 minimal medium: to the 700 mL of sterile water, add 200 mL of M9 mineral salt solution, 2 mL of 1M MgSO4 , and 100 mL of 1M CaCl2. Adjust volume to 1000 mL with sterile distilled water.

## Primers and probes

**TABLE S1** Primers and probes

| Name | Type | Sequence (5′-3′) | Target | Modifications | Reference |
|---|---|---|---|---|---|
| stn_1099F | primer | TAC CTG AAC GCT ATT CAT GCG ATT | stn | N/A | draft qPCR method |
| stn_1099F2 | primer | TAT CTG AAT GCT ATT CAC GCG ATT | stn | N/A | draft qPCR method |
| stn_1265R | primer | CCG TCA GCT TTS GTC GTA AAR TAA | stn | N/A | draft qPCR method |
| invA_1869F | primer | CCT CCG CTA ATT TGA TGG ATC TCA | invA | N/A | draft qPCR method |
| invA_1999R | primer | CGG AAA ACG ACC TTC AAT CAT TTT | invA | N/A | draft qPCR method |
| stn_1171P | probe | CGC GTT ATC ATC ACT GTT ACC GAT AGC G | stn | 5′ 6-FAM 3′ IABkFQ | draft qPCR method |
| invA_1962P | probe | CGG ACA TCG ACA GAC GTA AGG AGG ACA A | invA | 5′ HEX 3′ IABkFQ | draft qPCR method |
| 16S-F_341F | primer | CCTACGGGNGGCWGCAG | 16S V3-V4 region | Illumina adapters + i7 indices | Klindworth, et al. (2013) |
| 16S-R-785R | primer | GACTACHVGGGTATCTAATCC | 16S V3-V4 region | Illumina adapters + i7 indices | Klindworth, et al. (2013) |

*6-FAM is 6-caboxyfluorescein; HEX is 7,2′,4′,5′,7-hexachloro-6-carboxy-fluorescein; IABkFQ is TAO Iowa Black® RQ-Sp quencher.*

**TABLE S2** qPCR thermal cycling program

| Time | Temperature (°C) | Cycles |
|---|---|---|
| 5 min | 95 | 1 |
| 10 s | 95 | |
| 15 s | 58 | 45 |
| 10 s | 72 | |
| 30 s | 37 | 1 |

## 16S Sequencing Library Preparation

**TABLE** S3  PCR reaction mixtures

| | Volume (µL) per reaction |
|---|---|
| Components | 16S V3-V4 |
| Sample DNA | 2.5 |
| Forward primer | 5 (1 µM) |
| Reverse primer | 5 (µM) |
| 2X Kapa HiFi HotStart ready mix | 12.5 |
| H2O | 0 |
| Total volume | 25 |

**TABLE** S4  PCR temperature profiles

| Time | Temperature (°C) | Cycles |
|---|---|---|
| 3 min | 95 | 1 |
| 30 s | 95 | |
| 30 s | 55 | 25 |
| 30 s | 72 | |
| 5 min | 72 | 1 |
| hold | 4 | 1 |

## Kraken 2 host databases

Host or matrix-derived sequencing reads were removed from shotgun sequencing datasets after classification by comparison to host databases. To make the custom kr2chicken database, the reference genome for *Gallus gallus* (GRCg6a; GenBank accession GCA_000002315.5) was downloaded and all fasta files were concatenated into a library.fna file. This was used to create a custom Kraken 2 database following Kraken 2 manual instructions:

```
#Download the NCBI taxonomy
kraken2-build --download-taxonomy --db kr2chicken

#Add the library.fna file to the database
kraken2-build --add-to-library library.fna --db kr2chicken

#Build the library
kraken2-build --build --db kr2chicken/ --threads 32
```

The Kraken 2 plant database was downloaded on December 8, 2022, following the Kraken 2 manual.

```
#Download NCBI taxonomy
kraken2-build --download-taxonomy --db kr2plant
#Download the pre-existing plant library
kraken2-build --download-library plant --db kr2plant
#Build the database
kraken2-build --build --db kr2plant
```

## Investigation of potential false-positive *Salmonella* reads in unspiked control samples

Primer sets were created to screen colonies from BPW enrichments of negative control samples using SigSeekr [1] as follows. Briefly, paired-end reads from the unspiked (negative control) BPW enrichments of feed samples (n=3), which were identified as *Salmonella*-derived via the above pipeline, were concatenated and assembled using MEGAHIT v1.2.9 [2] with default settings. Each of the 11 contigs in the output assembly were tested against the NCBI-nt database using MEGABLAST as described above. MEGABLAST resulted in hits to *Citrobacter* and *E. coli* for one of the assembled contigs, which was subsequently used as an exclusion sequence for SigSeekr along with the assembly for the spiked target *Salmonella* (CFIAFB20140150); all other contigs in the assembled metagenome were left in the inclusion group as megablast reports only included *Salmonella* hits. SigSeekr output three potential sequences for primer creation,

shown below. These sequences were used with Primer3 to create primer pairs for 106 to 117 bp amplicons (Table S5).

>sequence1 - hits *E. coli*, *Citrobacter*, etc
TCCTGATTGACAGCCGCGATACCGAGACCGACAGCCGCCTGGACGGTTTG
AGTGACGCTTTCAGTGTATTCCGCTGTCACAGCATCATGAACTGCGTCAG
CGTATGTCCTAAGGGGCTGAACCCGACGCGCGCCATCGGCCATATTAAGT
>sequence2 - hits *Salmonella* and *Citrobacter*
CTGTTGCCCAGCGCCCGCGCCGCAGCCACGTCGCTGATTTCGGTATTTGC
GTGGATGCCCGGTGGCAAAACCGCGGTGTCGCCAGCGCGCTGATCCGCAC
GATGATCGACATGTGCGATAACTGGCTGCGCGTCGACCGTATTGAGTTGA
>sequence3 - hits only *Salmonella*
AAGCTTCCCCGCCCCACCTTGTGGGGCGAAACGTCACAAACCTTCAGAAC
ATTCCCGTTATTGCAACCAACTTCCCCTATATAAAAACAATAACCATCTG
AATTTCATGGTTATTGCCACTTTAAGCTTTATCACCGTTTTTCCGTAAA

**TABLE** S5 Primers created from SigSeekr potential targets

| SigSeekr Target | Primers | | Blast Hits |
|---|---|---|---|
| sequence1 | F: CTGATTGACAGCCGCGATAC<br>R: CAGCCCCTTAGGACATACGC | 117 | *E. coli* and *Citrobacter* |
| sequence2 | F: AGCCACGTCGCTGATTTCG<br>R: GCAGCCAGTTATCGCACAT | 106 | *Salmonella* and *Citrobacter* |
| sequence3 | F: GGGCGAAACGTCACAAACC<br>R: ACGGTGATAAAGCTTAAAGTGGC | 115 | Only *Salmonella* |

To select a positive PCR control, a database was created using the three potential 150 bp sequences for primer creation output by SigSeekr then used to screen *Citrobacter* and *Salmonella* assemblies in the OLC-CFIA sequence collection using KMA v1.4.3 [3]. All three sequences mapped to all *Salmonella* and to a single *Citrobacter werkmanii* assembly. Other *Citrobacter* sp. in the collection only encoded two of the three SigSeekr sequences (sequence1 and sequence2, Table S5).

## Isolation of bacteria producing false-positive *Salmonella* hits in BPW enrichments

Frozen glycerol stock of BPW enrichment for feed sample negA was thawed. A 500 µL aliquot of thawed stock was transferred to 100 mL of fresh sterile BPW and incubated overnight at 37°C with 150 rpm shaking. Overnight culture was serially diluted in sterile BPW, and 100 µL of dilutions were spread plated on Universal Differential ChromoSelect Medium (UChrom) agar (MilliporeSigma, Ottawa, Canada) in triplicate. Agar plates were incubated overnight at 37°C. Using a sterile pick, 94 CFU were selected and patched onto a gridded UChrom plate then transferred to a well of a 96-well PCR plate containing 100 µL of 1 % Triton-X-100. The PCR plate containing CFUs was boiled for 10 minutes at 100°C. The 25 µL reaction mixture for each of the primer pairs in Table S5 contained 5 µL of the Triton-X-100 lysate, 12.5 µL of GoTaq Colorless mastermix (Promega, Ottawa, Canada), 6.75 µL of molecular grade water,and 0.3 µM of each primer (individual primer pairs, not as a multiplex). PCR was performed using a BioRad C1000 Touch Thermal Cycler with the following conditions: 2 min. At 95°C, 40 cycles of 30s denaturation at 95°C, 30s annealing at 60°C, 30s extension at 72°C, followed by a final elongation at 72°C for 5 min. PCR amplicons were analyzed using capillary electrophoresis on the QIAxcel DNA Screening system (QIAGEN,Toronto, Canada) using method AM320 with QX alignment markers 15/3000 bp and QX DNA size marker 100 - 2500 bp. Following PCR, four colonies were selected for WGS. Purple colonies were selected, as *Citrobacter* sp. appear as purple colonies on UChrom, and we hypothesized Kraken2 may be incorrectly reporting reads from *Citrobacter* species as *Salmonella*. Isolate DNA preparation and sequencing was conducted as described by Cooper et al. [4].

## R packages

Plotting and statistical analyses were carried out in R v4.2.3 [5] using Rstudio v2023.03.1 [6]. The packages used were tidyverse [7], reshape2 [8], egg [9], scales [10], ggpubr [11], here [12], qiime2r [13], and phyloseq [14].

## Supplementary references

## References

[1] Michael Knowles, Dominic Lambert, George Huszczynski, Martine Gauthier, and Burton W Blais. PCR for the specific detection of an escherichia coli o157: H7 laboratory control strain. *Journal of food protection*, 78(9):1738–1744, 2015.

[2] Dinghua Li, Chi-Man Liu, Ruibang Luo, Kunihiko Sadakane, and Tak-Wah Lam. MEGAHIT: an ultra-fast single-node solution for large and complex metagenomics assembly via succinct de bruijn graph. *Bioinformatics*, 31(10):1674–1676, 2015.

[3] Philip TLC Clausen, Frank M Aarestrup, and Ole Lund. Rapid and precise alignment of raw reads against redundant databases with kma. *BMC bioinformatics*, 19:1–8, 2018.

[4] Ashley L Cooper, Andrew J Low, Adam G Koziol, Matthew C Thomas, Daniel Leclair, Sandeep Tamber, Alex Wong, Burton W Blais, and Catherine D Carrillo. Systematic evaluation of whole genome sequence-based predictions of Salmonella serotype and antimicrobial resistance. *Frontiers in microbiology*, 11:549, 2020.

[5] R Core Team. *R: A Language and Environment for Statistical Computing*. R Foundation for Statistical Computing, Vienna, Austria, 2021. URL https://www.R-project.org/.

[6] RStudio Team. *RStudio: Integrated Development Environment for R*. RStudio, PBC., Boston, MA, 2020. URL http://www.rstudio.com/.

[7] Hadley Wickham, Mara Averick, Jennifer Bryan, Winston Chang, Lucy D'Agostino McGowan, Romain François, Garrett Grolemund, Alex Hayes, Lionel Henry, Jim Hester, Max Kuhn, Thomas Lin Pedersen, Evan Miller, Stephan Milton Bache, Kirill Müller, Jeroen Ooms, David Robinson, Dana Paige Seidel, Vitalie Spinu, Kohske Takahashi, Davis Vaughan, Claus Wilke, Kara Woo, and Hiroaki Yutani. Welcome to the tidyverse. *Journal of Open Source Software*, 4(43):1686, 2019. doi: 10.21105/joss.01686.

[8] Hadley Wickham. Reshaping data with the reshape package. *Journal of Statistical Software*, 21(12):1–20, 2007. URL http://www.jstatsoft.org/v21/i12/.

[9] Baptiste Auguie. *egg: Extensions for 'ggplot2': Custom Geom, Custom Themes, Plot Alignment, Labelled Panels, Symmetric Scales, and Fixed Panel Size*, 2019. URL https://CRAN.R-project.org/package=egg. R package version 0.4.5.

[10] Hadley Wickham and Dana Seidel. *scales: Scale Functions for Visualization*, 2022. URL https://CRAN.R-project.org/package=scales. R package version 1.2.1.

[11] Alboukadel Kassambara. *ggpubr: 'ggplot2' Based Publication Ready Plots*, 2023. URL https://CRAN.R-project.org/package=ggpubr. R package version 0.6.0.

[12] Kirill Müller. *here: A Simpler Way to Find Your Files*, 2020. URL https://CRAN.R-project.org/package=here. R package version 1.0.1.

[13] Jordan E Bisanz. qiime2r: Importing QIIME2 artifacts and associated data into r sessions. v0.99, 2018. URL https://github.com/jbisanz/qiime2R.

[14] Paul J McMurdie and Susan Holmes. phyloseq: an R package for reproducible interactive analysis and graphics of microbiome census data. *PloS one*, 8(4):e61217, 2013.

# Supplementary figures

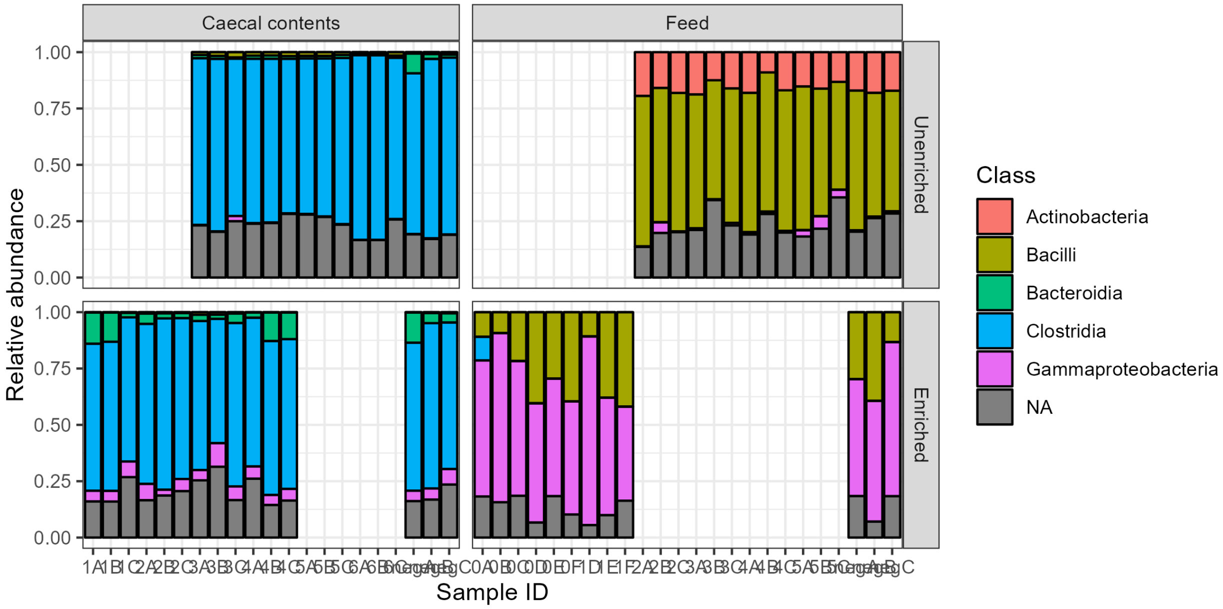


**FIG** S1 Community composition of samples from tested matrices without vs. with enrichment, based on sequencing of the 16S-V3V4 region. Sections of bars are coloured by taxonomic class. NA represents the sum of taxa that are < 0.1 % of the total community.

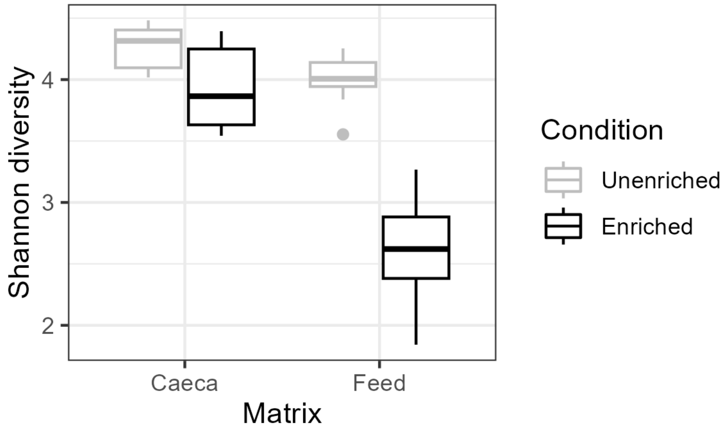


**FIG** S2 Alpha diversity measures of communities based on sequencing of the 16S-V3V4 region.

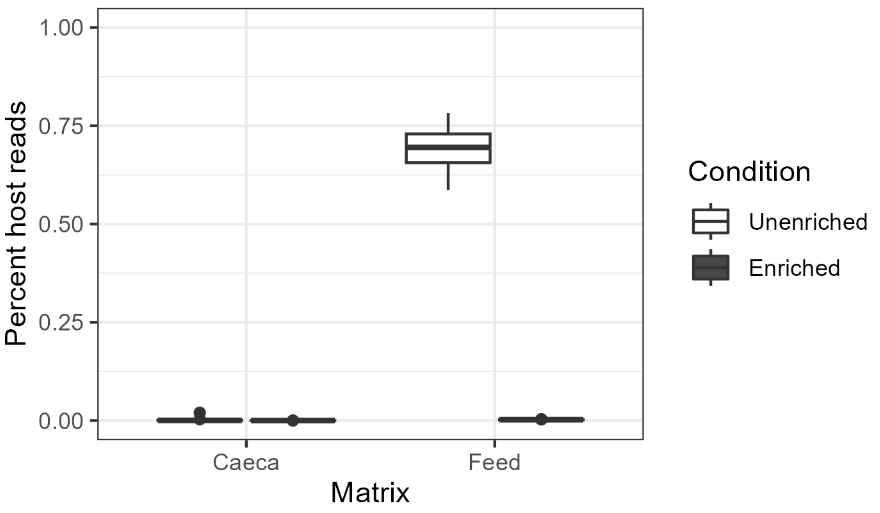


**FIG** S3 Proportion of reads in shotgun sequencing datasets that matched to host databases (caecal samples: chicken genome database; feed samples: plant database).

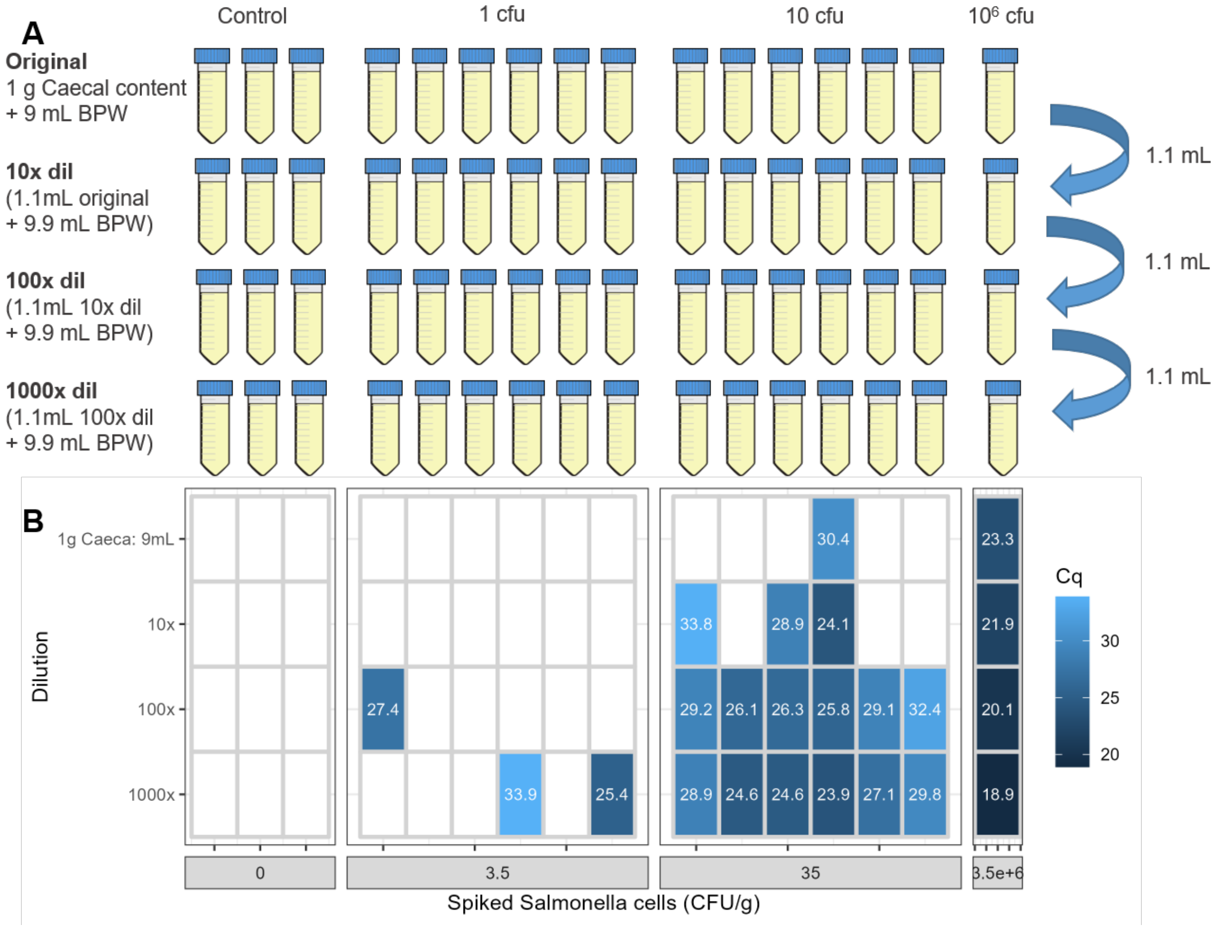

**FIG** S4 A) Schematic for the caecal contents-BPW dilution experiment. Values on the left show the expected count of background microbiota from the caecal contents, and values along the top show the number of Salmonella cells spiked in. B) Cq values for the invA gene at various Salmonella spike-in levels and dilutions.

## Supplementary data tables

**TABLE** S6 Overview of Salmonella detection in caecal contents

| Spiked CFU/g | | | | Enriched | | | Unenriched | | |
|---|---|---|---|---|---|---|---|---|---|
| Expected | Actual | N | C.D. | qPCR | 16S | Shotgun | qPCR | 16S | Shotgun |
| $10^6$ | $5.9x10^5$ | 3 | 100 % | 100 % | N/A | 100 % | 100 % | 0 % | 100 % |
| $10^5$ | $5.9x10^4$ | 3 | 100 % | 100 % | N/A | 100 % | 100 % | 0 % | 100 % |
| $10^4$ | $5.9x10^3$ | 3 | 100 % | 100 % | N/A | 100 % | 100 % | 0 % | 0 % |
| $10^3$ | $5.9x10^2$ | 3 | 100 % | 100 % | 0 % | 67 % | 0 % | 0 % | 0 % |
| $10^2$ | $5.9x10^1$ | 3 | 33 % | 67 % | 0 % | 33 % | 0 % | N/A | 0 % |
| $10^1$ | 5.9 | 6 (3) | 0 % | 0 % | 0 % | 0 % | 0 % | N/A | 0 % |
| 1 | 0.6 | 6 (3) | 0 % | 0 % | 0 % | 0 % | 0 % | N/A | 0 % |
| 0 (ctrl) | | 3 | 0 % | 0 % | 0 % | 0 % | 0 % | 0 % | 0 % |

*Percent values are the percent of replicates in which Salmonella was detected;*
*N: number of replicates. Number selected for 16S and shotgun sequencing are in brackets;*
*C.D: culture-dependent* *qPCR results shown are for 40 cycle threshold*

**TABLE** S7 Overview of *Salmonella* detection in feed

| Spiked CFU/10g | | | | Enriched | | | Unenriched | | |
|---|---|---|---|---|---|---|---|---|---|
| Expected | Actual | N | C.D. | qPCR | 16S | Shotgun | qPCR | 16S | Shotgun |
| $10^6$ | $7.5\ x10^5$ | 3 (0,3) | 100 % | 100 % | N/A | N/A | 100 % | N/A | 100 % |
| $10^5$ | $7.5\ x10^4$ | 3 (0,3) | 100 % | 100 % | N/A | N/A | 100 % | 100 % | 100 % |
| $10^4$ | $7.5\ x10^3$ | 3 (0,3) | 100 % | 100 % | N/A | N/A | 100 % | 100 % | 100 % |
| $10^3$ | $7.5\ x10^2$ | 3 (3,3) | 100 % | 100 % | N/A | 100 % | 100 % | 0 % | 100 % |
| $10^2$ | 75 | 3 (3,3) | 100 % | 100 % | N/A | 100 % | 0 % | 0 % | 100 % |
| $10^1$ | 7.5 | 6 (3,0) | 100 % | 100 % | 100 % | 100 % | 0 % | N/A | N/A |
| 1 | 0.75 | 6 (6,0) | 66 % | 50 % | 50 % | 100 % | 0 % | N/A | N/A |
| 0 (ctrl) | 0 | 3 (3,3) | 0 % | 66 %* | 0 % | 100 % | 0 % | 0 % | 100 % |

*Percent values are the percent of replicates in which Salmonella was detected;*

*C.D: culture-dependent;*

*N: number of replicates. Number selected for 16S and shotgun sequencing are in brackets, in the format (enriched,unenriched);*

*N/A: samples at this combination of spiking/condition were not selected for sequencing*

**Positives based on amplification of only one target gene fragment in multiplex qPCR*

*qPCR results shown are for 40 cycle threshold*

**TABLE** S8 Shotgun sequencing reads identified as *Salmonella* in unspiked feed samples.

| Replicate | Condition | Total non-host | Salmonella hits* | NCBI** |
|---|---|---|---|---|
| A | Unenriched | $3.27x10^{7}$ | 0 | NA |
| B | Unenriched | $2.64x10^{7}$ | 3 | 1 |
| C | Unenriched | $2.38x10^{7}$ | 1 | 1 |
| A | Enriched | $8.82x10^{7}$ | 1091 | 675 |
| B | Enriched | $9.94x10^{7}$ | 32 | 23 |
| C | Enriched | $7.76x10^{7}$ | 30 | 5 |

** Reads identified as Salmonella-derived according to the shotgun sequencing analysis pipeline*

** Reads identified as Salmonella-derived and matching only to Salmonella in NCBI-nt*

**TABLE** S9: Raw read counts from the three sequencing methods used in this study

| SampleID | Matrix | Condition | Shotgun* | 16S V3-V4** |
|---|---|---|---|---|
| 0A | Caeca | Enriched | $9.4x10^{7}$ | NA |
| 0A | Caeca | Unenriched | $7.5x10^{7}$ | NA |
| 0B | Caeca | Enriched | $6.4x10^{7}$ | NA |
| 0B | Caeca | Unenriched | $5.2x10^{7}$ | NA |
| 0C | Caeca | Enriched | $4.7x10^{7}$ | NA |
| 0C | Caeca | Unenriched | $5.3x10^{7}$ | NA |
| 1A | Caeca | Enriched | $7.1x10^{7}$ | $9.8x10^{4}$ |
| 1A | Caeca | Unenriched | $5.9x10^{7}$ | NA |
| 1B | Caeca | Enriched | $5.8x10^{7}$ | $1.1x10^{5}$ |
| 1B | Caeca | Unenriched | $6.5x10^{7}$ | NA |
| 1C | Caeca | Enriched | $6.0x10^{7}$ | $1.0x10^{5}$ |
| 1C | Caeca | Unenriched | $6.7x10^{7}$ | NA |
| 2A | Caeca | Enriched | $6.9x10^{7}$ | $9.0x10^{4}$ |
| 2A | Caeca | Unenriched | $5.2x10^{7}$ | NA |
| 2B | Caeca | Enriched | $5.4x10^{7}$ | $1.4x10^{5}$ |

**Table S9 continued from previous page**

| SampleID | Matrix | Condition | Shotgun* | 16S V3-V4** |
|---|---|---|---|---|
| 2B | Caeca | Unenriched | $4.9x10^7$ | NA |
| 2C | Caeca | Enriched | $5.8x10^7$ | $1.2x10^5$ |
| 2C | Caeca | Unenriched | $5.3x10^7$ | NA |
| 3A | Caeca | Enriched | $9.0x10^7$ | $1.2x10^5$ |
| 3A | Caeca | Unenriched | $6.4x10^7$ | $9.8x10^4$ |
| 3B | Caeca | Enriched | $8.8x10^7$ | $1.4x10^5$ |
| 3B | Caeca | Unenriched | $5.3x10^7$ | $1.1x10^5$ |
| 3C | Caeca | Enriched | $6.2x10^7$ | $1.1x10^5$ |
| 3C | Caeca | Unenriched | $5.2x10^7$ | $1.1x10^5$ |
| 4A | Caeca | Enriched | $5.3x10^7$ | $9.8x10^4$ |
| 4A | Caeca | Unenriched | $5.9x10^7$ | $1.4x10^5$ |
| 4B | Caeca | Enriched | $4.9x10^7$ | $1.1x10^5$ |
| 4B | Caeca | Unenriched | $6.1x10^7$ | $1.3x10^5$ |
| 4C | Caeca | Enriched | $4.2x10^7$ | $1.2x10^5$ |
| 4C | Caeca | Unenriched | $5.9x10^7$ | $1.5x10^5$ |
| 5A | Caeca | Enriched | $4.4x10^7$ | NA |
| 5A | Caeca | Unenriched | $5.6x10^7$ | $1.5x10^5$ |
| 5B | Caeca | Enriched | $9.1x10^7$ | NA |
| 5B | Caeca | Unenriched | $8.4x10^7$ | $1.2x10^5$ |
| 5C | Caeca | Enriched | $9.1x10^7$ | NA |
| 5C | Caeca | Unenriched | $5.6x10^7$ | $1.2x10^5$ |
| 6A | Caeca | Enriched | $4.7x10^7$ | NA |
| 6A | Caeca | Unenriched | $5.1x10^7$ | $1.4x10^5$ |
| 6B | Caeca | Enriched | $4.8x10^7$ | NA |
| 6B | Caeca | Unenriched | $5.7x10^7$ | $1.5x10^5$ |
| 6C | Caeca | Enriched | $4.7x10^7$ | NA |
| 6C | Caeca | Unenriched | $8.7x10^7$ | $9.9x10^4$ |
| negA | Caeca | Enriched | $5.9x10^7$ | $1.0x10^5$ |
| negA | Caeca | Unenriched | $6.9x10^7$ | $1.5x10^5$ |

**Table S9 continued from previous page**

| SampleID | Matrix | Condition | Shotgun* | 16S V3-V4** |
|---|---|---|---|---|
| negB | Caeca | Enriched | $4.9x10^7$ | $9.1x10^4$ |
| negB | Caeca | Unenriched | $7.2x10^7$ | $1.6x10^5$ |
| negC | Caeca | Enriched | $5.0x10^7$ | $1.0x10^5$ |
| negC | Caeca | Unenriched | $4.3x10^7$ | $1.3x10^5$ |
| 0A | Feed | Enriched | $7.9x10^7$ | $9.7x10^4$ |
| 0B | Feed | Enriched | $5.7x10^7$ | $9.3x10^4$ |
| 0C | Feed | Enriched | $7.7x10^7$ | $7.8x10^4$ |
| 0D | Feed | Enriched | $7.6x10^7$ | $9.2x10^4$ |
| 0E | Feed | Enriched | $8.2x10^7$ | $6.2x10^4$ |
| 0F | Feed | Enriched | $1.0x10^8$ | $9.4x10^4$ |
| 1B | Feed | Enriched | $7.2x10^7$ | NA |
| 1C | Feed | Enriched | $8.8x10^7$ | NA |
| 1D | Feed | Enriched | NA | $8.2x10^4$ |
| 1E | Feed | Enriched | NA | $9.6x10^4$ |
| 1F | Feed | Enriched | $8.4x10^7$ | $1.0x10^5$ |
| 2A | Feed | Enriched | $1.0x10^8$ | NA |
| 2A | Feed | Unenriched | $6.5x10^7$ | $6.3x10^4$ |
| 2B | Feed | Enriched | $7.7x10^7$ | NA |
| 2B | Feed | Unenriched | $7.3x10^7$ | $1.4x10^5$ |
| 2C | Feed | Enriched | $8.8x10^7$ | NA |
| 2C | Feed | Unenriched | $7.3x10^7$ | $1.3x10^5$ |
| 3A | Feed | Enriched | $9.5x10^7$ | NA |
| 3A | Feed | Unenriched | $7.1x10^7$ | $1.2x10^5$ |
| 3B | Feed | Enriched | $8.3x10^7$ | NA |
| 3B | Feed | Unenriched | $6.5x10^7$ | $1.4x10^5$ |
| 3C | Feed | Enriched | $9.4x10^7$ | NA |
| 3C | Feed | Unenriched | $8.0x10^7$ | $1.7x10^5$ |
| 4A | Feed | Unenriched | $5.6x10^7$ | $1.4x10^5$ |
| 4B | Feed | Unenriched | $5.0x10^7$ | $1.6x10^5$ |

**Table S9 continued from previous page**

| SampleID | Matrix | Condition | Shotgun* | 16S V3-V4** |
|---|---|---|---|---|
| 4C | Feed | Unenriched | $7.5x10^7$ | $1.2x10^5$ |
| 5A | Feed | Unenriched | $6.7x10^7$ | $9.1x10^4$ |
| 5B | Feed | Unenriched | $5.8x10^7$ | $1.1x10^5$ |
| 5C | Feed | Unenriched | $6.5x10^7$ | $1.2x10^5$ |
| 6A | Feed | Unenriched | $6.2x10^7$ | NA |
| 6B | Feed | Unenriched | $7.2x10^7$ | NA |
| 6C | Feed | Unenriched | $7.7x10^7$ | NA |
| negA | Feed | Enriched | $8.9x10^7$ | $9.9x10^4$ |
| negA | Feed | Unenriched | $9.4x10^7$ | $1.2x10^5$ |
| negB | Feed | Enriched | $1.0x10^8$ | $7.6x10^4$ |
| negB | Feed | Unenriched | $6.5x10^7$ | $2.1x10^5$ |
| negC | Feed | Enriched | $7.8x10^7$ | $8.1x10^4$ |
| negC | Feed | Unenriched | $8.0x10^7$ | $1.3x10^5$ |

*NA: Sequencing not performed.*

** Sequenced on an Illumina NovaSeq6000*

*** Sequenced on an Illumina MiSeq*
